# Expression and Role of β3-Adrenergic Receptor during the Differentiation of 3T3-L1 Preadipocytes into Adipocytes

**DOI:** 10.3390/biology11050772

**Published:** 2022-05-18

**Authors:** Amir Roshanzadeh, Anil Kumar Yadav, Sai-Prasad Pydi, Takefumi Kimura, Byeong-Churl Jang

**Affiliations:** 1 Department of Molecular Medicine, College of Medicine, Keimyung University, 1095 Dalgubeoldaero, Dalseo-gu, Daegu 42601, Korea; amirroushanzadeh@gmail.com (A.R.); yadav127@umn.edu (A.K.Y.); 2 The Hormel Institute, University of Minnesota, Austin, MN 55912, USA; 3 Molecular Signaling Section, Laboratory of Bioorganic Chemistry, National Institute of Diabetes and Digestive and Kidney Diseases, Bethesda, MD 20892, USA; saiprasad.pydi@nih.gov (S.-P.P.); takefumi.kimura@nih.gov (T.K.)

**Keywords:** β3AR, 3T3-L1, C/EBP-α, PPAR-γ, perilipin A

## Abstract

**Simple Summary:**

The β3-adrenergic receptor (β3-AR) has long been viewed as a potential therapeutic target for dealing with obesity. Although the lipolytic and thermogenic role of β3-AR in brown/beige adipocytes is well defined, the β3-AR’s adipogenic role in white adipocytes remains unclear at present. In this study, we investigated the expression and function of β3-AR in 3T3-L1 murine white preadipocytes. Knockdown of β3-AR led to less lipid accumulation and triglyceride (TG) content as well as less expression and phosphorylation levels of CCAAT/enhancer-binding protein-α (C/EBP-α) and peroxisome proliferator-activated receptor-γ (PPAR-γ) during 3T3-L1 preadipocyte differentiation. These findings reveal that the β3-AR inhibitor or antagonist could be a promising candidate for potential preventive and therapeutics against obesity.

**Abstract:**

β3-adrenergic receptor (β3-AR) is expressed predominantly in mature white and brown/beige adipocytes. Although the lipolytic and thermogenic role of β3-AR in brown/beige adipocytes is well defined, the adipogenic role of β3-AR in white adipocytes remains unclear at present. In this study, we investigated the expression and function of β3-AR in differentiating 3T3-L1 cells, murine white preadipocytes. Of note, the expression of β3-AR at the protein and mRNA levels was highly induced in a time-dependent manner during 3T3-L1 preadipocyte differentiation. Interestingly, the results of the pharmacological inhibition study demonstrated the roles of p38 MAPK and PKC in the induction of β3-AR expression in differentiating 3T3-L1 cells. Knockdown of β3-AR led to less lipid accumulation and triglyceride (TG) content during 3T3-L1 preadipocyte differentiation with no cytotoxicity. Furthermore, knockdown of β3-AR resulted in a decrease in not only expression levels of CCAAT/enhancer-binding protein-α (C/EBP-α), peroxisome proliferator-activated receptor-γ (PPAR-γ), fatty acid synthase (FASN), perilipin A, and leptin but also phosphorylation levels of signal transducer and activator of transcription-5 (STAT-5) during 3T3-L1 preadipocyte differentiation. In summary, these results demonstrate firstly that β3-AR expression is highly up-regulated in p38 MAPK and PKC-dependent manners, and the up-regulated β3-AR plays a crucial role in lipid accumulation in differentiating 3T3-L1 cells, which is mediated through control of expression and phosphorylation levels of C/EBP-α, PPAR-γ, STAT-5, FASN, and perilipin A.

## 1. Introduction

Obesity is defined as abnormal fat accumulation in the human body. Obesity has now become a global pandemic, based on the fact that it is highly associated with the development of many human chronic diseases such as type 2 diabetes, hypertension, and cancer [1,2]. Obesity is an enlargement of adipose tissue to store excess energy intake. Hyperplasia (increase in number) and hypertrophy (increase in size) of adipocytes are two possible mechanisms for the induction of obesity [3]. A wealth of information illustrates that excessive preadipocyte differentiation (hypertrophy) leads to excessive fat (mainly in the form of triglyceride (TG)) accumulation in adipocytes and resultantly in the development of obesity [4,5]. Increasing evidence suggests that G-protein coupled receptors (GPCRs), including β3-adrenergic receptor (β3-AR), regulate fat storage in the adipose tissues and adipocytes [6,7]. β3-AR is expressed predominantly in mature (or differentiated) white and brown/beige adipocytes [8]. β3-AR is currently regarded as one of the potential anti-obesity targets, based on the fact that β3-AR selective agonists can reduce fat storage in mature white and brown/beige adipocytes by stimulating lipolysis and thermogenesis [9,10]. However, as of now, the expression and role of β3-AR in white preadipocyte differentiation remain unclear.

Preadipocyte differentiation also called adipogenesis, is a multistep process that occurs in the form of cellular, morphological, and biochemical changes [11]. The process converts fibroblast-like preadipocytes into mature adipocytes that are filled with many lipid droplets (LDs) [12,13]. Multiple adipogenic transcription factors, including CCAAT/enhancer-binding proteins (C/EBPs), peroxisome proliferator-activated receptors (PPARs), and signal transducer and activator of transcription (STAT) proteins, play a pivotal role in preadipocyte differentiation [14,15]. Preadipocyte differentiation also involves lipogenesis and LDs maturation/stabilization, which requires fatty acid synthase (FASN) and perilipin A [16]. Increasing evidence further indicates that many protein kinases, including extracellular signal-regulated protein kinase-1/2 (ERK-1/2), p38 mitogen-activated protein kinase (MAPK), protein kinase C (PKC), cAMP-activated protein kinase (AMPK), and protein kinase A (PKA), participate in the regulation of preadipocyte differentiation [17,18,19,20].

RNA sequencing is a technique for identifying differentially expressed RNAs in cells or tissues by high-throughput sequencing [21]. In order to see the transcriptional events regulating white preadipocyte differentiation and function, we have recently performed RNA sequencing at the preadipocyte (D0), differentiating adipocyte (D2), and differentiated adipocyte (D7) stages of 3T3-L1 cells, a murine white preadipocyte, and found that multiple RNAs including β3-AR are highly up-regulated in the process [22]. At present, the regulation of β3-AR expression in white preadipocyte differentiation also remains elusive. The gene encoding β3-AR in mice and humans was found to be highly identical in their transmembrane domains [23]. Therefore, in this study, we investigated the expression regulation and role of β3-AR in 3T3-L1 preadipocyte differentiation. We demonstrate that β3-AR expression is highly up-regulated in p38 MAPK and PKC-dependent manners, and knockdown of β3-AR causes less lipid accumulation and TG content during 3T3-L1 preadipocyte differentiation through control of the expression and phosphorylation levels of C/EBP-α, PPAR-γ, STAT-5, FASN, and perilipin A.

## 2. Materials and Methods

### 2.1. Chemicals and Antibodies

Control shRNA plasmid (Cat. No. sc-108060) and β3-AR shRNA plasmid (Cat. No. sc-39869) were purchased from Santa Cruz Biotechnology (Delaware, CA, USA). PD98059, SB203580 (Cat. No. BML-E1294) was purchased from Enzo (Farmingdale, NY, USA). SP600125 (Cat. No. 420119) was from Calbiochem (Madison, WI, USA). LY294002 (Cat. No. ST-420) and GF109203X (Cat. No. EI-246) were purchased from Biomol (Hamburg, Germany). 3-isobutyl-1-methylxanthine (IBMX), dexamethasone, and insulin were bought from Sigma (St. Louis, MO, USA). Primary antibodies specific for anti-β3-AR, anti-C/EBP-α, and anti-PPAR-γ, anti-actin, anti-phospho (p)-STAT-3 (Y705), anti-STAT-3, anti-phospho (p)-STAT-5 (Y694), and anti-STAT-5 were purchased from Santa Cruz Biotechnology (Delaware, CA, USA). Anti-perilipin A antibody was purchased from BioVision (Milpitas, CA, USA). The anti-FASN antibody was purchased from BD Bioscience (San Jose, CA, USA). Super Signal™ West Pico PLUS Enhanced chemiluminescence (ECL) (Cat. No. 34080) was purchased from Thermo Scientific (Waltham, MA, USA). All Plasticware, including 6-well, 24-well plates, 60 mm, and 100 mm cell culture dishes, were obtained from SPL Life Sciences (Pocheon-si, Korea).

### 2.2. Cell Culture and Differentiation

3T3-L1 preadipocytes (ATCC, Manassas, VA, USA) were cultured in Dulbecco’s Modified Eagle’s Medium (DMEM, Welgene, Daegu, Korea) supplemented with 10% heat-inactivated fetal calf serum (FCS, Gibco, Grand Island, NY, USA) and 1% penicillin/streptomycin (Welgene, Daegu, Korea) at 37 °C in a humidified atmosphere of 5% CO_2_. 3T3-L1 preadipocytes were grown up to the contact inhibition stage and remained in the post-confluent stage for 2 days (D0). Differentiation was induced by changing the medium to DMEM supplemented with 10% FBS (Welgene, Daegu, Korea) plus a cocktail of hormones (MDI) that include 0.5 mM IBMX (M) (Sigma), 0.5 µM dexamethasone (D) (Sigma), and 5 µg/mL insulin (I) (Sigma) for 2 days (D2). Cells were switched to DMEM supplemented with 10% FBS and 5 µg/mL insulin for additional 3 days (D5). Cells were then fed every other day with DMEM supplemented with 10% FBS for further 3 days (D8).

### 2.3. β3-AR Short-Hairpin RNA (shRNA) Transfection

3T3-L1 preadipocytes seeded into 6-well plates. After overnight incubation, cells were transfected with control or β3-AR shRNA using LipofectamineTM 2000 (Invitrogen, Waltham, MA, USA) for 6 h. The culture medium from the transfected cells was removed and refreshed with DMEM containing 10% FCS, followed by incubation for 48 h. Differentiation of the control or β3-AR shRNA-transfected 3T3-L1 cells was induced as per the above-mentioned differentiation conditions.

### 2.4. Oil Red O Staining

On D8 of differentiation, control or β3-AR stably knock-down 3T3-L1 cells were washed with phosphate-buffered saline (PBS) and fixed with 10% formaldehyde for 2 h at room temperature (RT). Next, cells were washed with 60% isopropanol and dried at RT. Cells were then stained with Oil Red O working solution (Sigma; St. Louis, MO, USA) for 1 h at RT and washed with deionized water. Stained LDs in control or β3-AR knock-down 3T3-L1 cells were visualized under the light microscope (Nikon, TS100, Tokyo, Japan).

### 2.5. Cell Survival Assay

Control or β3-AR shRNA-transfected 3T3-L1 preadipocytes were seeded in a 24-well plate and grown under the above-mentioned differentiation. On D8 of differentiation, cell counts were performed by staining control or β3-AR knock-down 3T3-L1 cells with 0.4% trypan blue dye. The cell count analysis was performed in triplicates. Data are mean ± standard error (SE) of three independent experiments.

### 2.6. Measurement of Intracellular TG

On D8 of differentiation, intracellular TG content in control or β3-AR shRNA-transfected 3T3-L1 cells were quantified using AdipoRed assay reagent kit (Lonza, Basel, Switzerland) according to the manufacturer’s protocol. Fluorescence intensity was quantified with excitation and emission at 485 and 572 nm, respectively, using Victor3 (Perkin Elmer, Boston, MA, USA).

### 2.7. Preparation of Whole-Cell Lysates

At the designated time points, 3T3-L1 cells were lysed in RIPA buffer (Sigma) supplemented with a proteinase inhibitor cocktail (1×). The cell lysates were centrifuged at 12,074× *g* for 20 min at 4 °C. The supernatant was saved, and its protein concentration was determined using bicinchoninic acid (BCA) protein assay kit (Thermo Scientific, Rockford, IL, USA).

### 2.8. Immunoblot Analysis

Proteins (50 µg) were loaded and run in 10% of SDS-polyacrylamide gel electrophoresis (SDS-PAGE). After separation of proteins, transferred onto polyvinylidene difluoride membrane (PVDF, Millipore, Bedford, MA, USA) and then blocked with 5% (*w*/*v*) skim milk in TBST for 1 h at room temperature. Membranes were incubated with specific antibodies at 4 °C. Later, membranes were rinsed with TBST buffer and further incubated with anti-goat IgG or anti-mouse IgG, or anti-rabbit IgG coupled with horseradish peroxidase for 2 h at RT. Next, membranes were rinsed three times with TBST and developed with enhanced chemiluminescence (ECL) reagents. Actin expression levels were used as an equal protein loading control.

### 2.9. Quantitative Real-Time RT-PCR

Total cellular RNA from Control or β3-AR shRNA-transfected 3T3-L1 cells was isolated with the RNAiso Plus (Takara, Kusatsu, Shiga, Japan). Three micrograms of total RNA were reverse transcribed using a ran-dom hexadeoxynucleotide primer and reverse transcriptase. Single-stranded cDNA was amplified by PCR with the following primers: β3-AR sense 5′-CCAGTCCCTGCCTATGTTTGT-3′; anti-sense 5′-GATGGTCCAAGATGGTGCTT-3′; C/EBP-α sense 5′-TTACAACAGGCCAGGTTTCC-3′; anti-sense 5′-GGCTGGCGACATACAGTACA-3′; PPAR-γ sense 5′-AGGCCGAGAAGGAGAAGCTGTTG-3′; anti-sense 5′-TGGCCACCTCTTTGCTCTGCTC-3′; FASN sense 5′-TTGCTGGCACTACAGAATGC-3′; anti-sense 5′-AACAGCCTCAGAGCGACAAT-3′; perilipin A sense 5′-C ACTCTCTGGCCATGTGGA-3′; anti-sense 5′-AGAGGCTGCCAGGTTGTG-3′; leptin sense 5′-GACACCAAAACCCTCAT-3′; anti-sense 5′-CAGTGTCTGGTCCATCT-3′; 18S rRNA sense 5′-CCATCCAATCGGTAGTAGCG-3′; anti-sense 5′-GTAACCCGTTGAACCCCATT-3′. SYBR Green PCR Master Mix (Takara, Kusatsu, Japan) was then analyzed with the LightCyclerâ96 Machine (Roche, Mannheim, Germany). All reactions were performed in triplicate for each sample. Quantitation was performed by the comparative Ct (2e Ct) method. The Ct value for each sample was normalized by the value for 18S rRNA.

### 2.10. Statistical Analyses

Numerical data are expressed as the mean ± S.D. for at least three independent experiments. We employed Student’s *t*-test and one-way ANOVA to analyze the statistical difference between two or more groups. *p* < 0.05 was considered statistically significant.

## 3. Results

### 3.1. β3-AR Expression Is Elevated in a Time-Dependent Manner during 3T3-L1 Preadipocyte Differentiation

**Figure 1:**
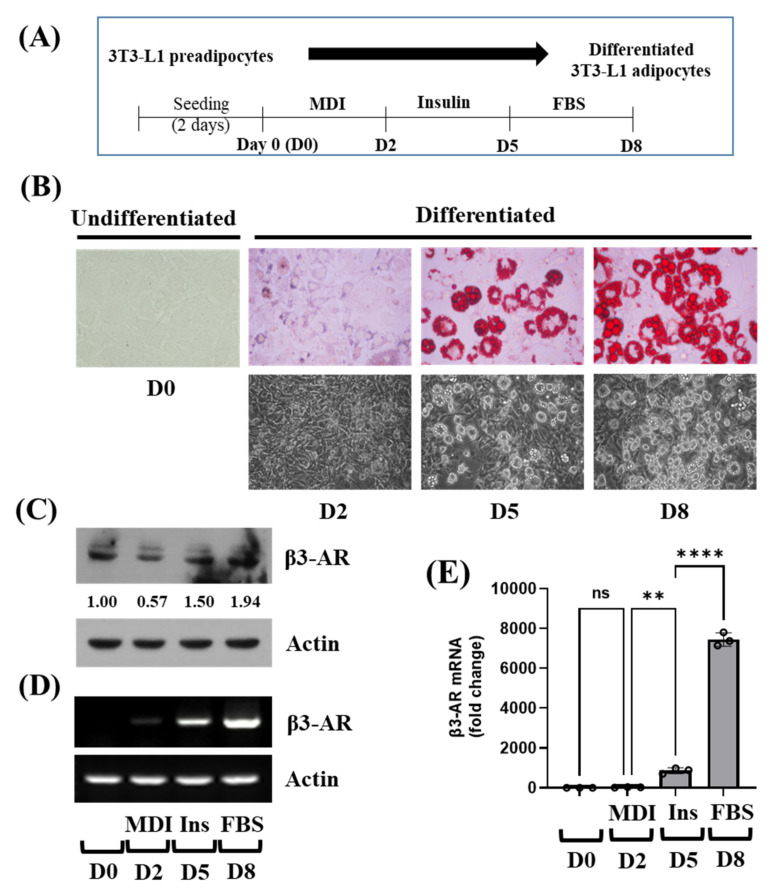
Lipid accumulation and expression of β3-AR at the protein and mRNA levels during 3T3-L1 preadipocyte differentiation. (**A**) The scheme for 3T3-L1 preadipocyte differentiation. (**B**) Measurement of lipid droplets (LDs) accumulation on day 0 (D0), D2, D5, and D8 of 3T3-L1 preadipocyte differentiation by Oil Red O staining (upper panels) and by phase-contrast image (lower panels). (**C**) 3T3-L1 preadipocytes were differentiated with an induction medium containing MDI, insulin, and FBS, and harvested at D0, D2, D5, and D8, respectively. At each time point, whole-cell lysates were prepared and analyzed by immunoblot analysis with respective antibodies. Relative intensities were measured by ImageJ software (version 1.8.0; National Institutes of Health). (**D**–**E**) 3T3-L1 preadipocytes were differentiated with an induction medium containing MDI, insulin, and FBS, and harvested at D0, D2, D5, and D8, respectively. At each time point, total cellular RNA was extracted and analyzed by RT-PCR (**D**) or real-time qPCR (**E**) with respective primers. In (**E**), Error bars are indicated as mean ± SD. ** *p* < 0.01; **** *p* < 0.0001; ns, not significant, calculated by one-way ANOVA with Sidak’s multiple comparison test.

The experimental protocol for the differentiation of 3T3-L1 preadipocytes into adipocytes is depicted in Figure 1A. Intracellular lipid accumulation during 3T3-L1 preadipocyte differentiation was primarily investigated using Oil Red O staining. There was a high accumulation of intracellular lipid droplets (LDs) in 3T3-L1 cells on D8 of differentiation, compared with undifferentiated cells (Figure 1B, upper panels). The intracellular LDs accumulation in 3T3-L1 cells on D8 of differentiation was also confirmed by phase-contrast microscopy (Figure 1B, lower panels). Next, we analyzed the expression levels of β3-AR protein during the differentiation of 3T3-L1 preadipocyte into adipocyte using Western blotting. As shown in Figure 1C, there were substantial levels of β3-AR protein in 3T3-L1 preadipocytes (on D0). However, levels of β3-AR protein expression rather slightly decreased on D2 of 3T3-L1 preadipocyte differentiation, but its level increased on D5 and D8. Next, we measured the expression levels of β3-AR mRNA during 3T3-L1 preadipocyte differentiation using RT-PCR and qPCR analyses. As shown in Figure 1D and E, there was a marked increase in β3-AR mRNA expression levels on D8 of differentiation compared with undifferentiated cells, suggesting the possibility of β3-AR being involved in the adipogenesis and the formation of LDs in 3T3-L1 cells.

### 3.2. cAMP, p38 MAPK, and PKC Are Crucial for the Induction of β3-AR mRNA during 3T3-L1 Preadipocyte Differentiation

**Figure 2:**
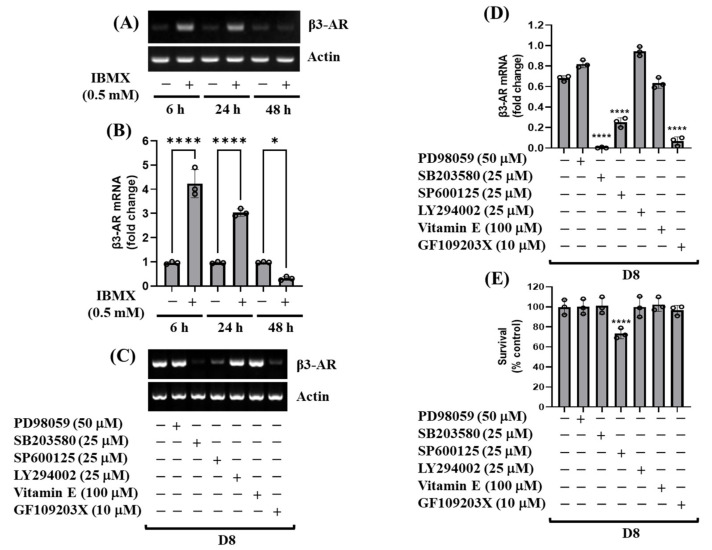
Effects of IBMX, PD98059, SB203580, SP600125, LY294002, vitamin E or GF109203X on mRNA expression of β3-AR in 3T3-L1 preadipocytes and differentiating 3T3-L1 cells. (**A**,**B**) 3T3-L1 preadipocytes were grown in the absence (control; 0.1% DMSO) or presence of IBMX (0.5 mM) at designated times. At each time point, total cellular RNA was extracted and analyzed by RT-PCR (**A**) and real-time qPCR (**B**) with respective primers. Error bars are indicated as mean ± SD. * *p* < 0.1; **** *p* < 0.0001; calculated by one-way ANOVA with Sidak’s multiple comparison test. (**C**–**E**) 3T3-L1 preadipocytes were differentiated with an induction medium containing MDI, insulin, and FBS in the absence (control; 0.1% DMSO) or presence of PD98059 (a MEK-1/2 (ERK-1/2) inhibitor), SB203580 (a p38 MAPK inhibitor), SP600125 (a JNK-1/2 inhibitor), LY294002 (a PI3K/PKB inhibitor), vitamin E (an antioxidant) or GF109203X (a PKC inhibitor) at designated concentrations and harvested at day 8 (D8). Total cellular RNA was then extracted and analyzed by RT-PCR (**C**) and real-time qPCR (**D**) with respective primers. The number of live cells in control or β3-AR shRNA-transfected cells on D8 was counted by trypan blue dye exclusion. Data are mean ± SE of three independent experiments, each performed in triplicate. Error bars are indicated as mean ± SD. **** *p* < 0.0001; calculated by one-way ANOVA with Sidak’s multiple comparison test.

We next sought to explore which signaling factor(s) or mechanism(s) are responsible for the induction of β3-AR mRNA expression in 3T3-L1 preadipocytes as well as during 3T3-L1 preadipocyte differentiation. Of interest, as shown in Figure 2A,B, data of RT-PCR and qPCR analyses, respectively, demonstrated that treatment with IBMX, one of the early differentiation inducers, led to a significant induction of β3-AR mRNA expression particularly at 6 or 24 h in 3T3-L1 preadipocytes, following a sharp decline at 48 h. In addition, as shown in Figure 2C–E, the results of the pharmacological inhibition study with PD98059 (a MEK-1/2 (ERK-1/2) inhibitor), SB203580 (a p38 MAPK inhibitor), SP600125 (a JNK-1/2 inhibitor), LY294002 (a PI3K/PKB inhibitor), vitamin E (an antioxidant) or GF109203X (a PKC inhibitor), showed that treatment with either SB203580 or GF109203X vastly suppressed the induction of β3-AR mRNA expression on D8 of 3T3-L1 preadipocyte differentiation with no cytotoxicity. However, although treatment with SP600125 strongly suppressed the induction of β3-AR mRNA expression on D8 of 3T3-L1 preadipocyte differentiation, the drug was highly cytotoxic to these cells. Moreover, treatment with PD98059, LY294002, or vitamin E did not influence the induction of β3-AR mRNA expression on D8 of 3T3-L1 preadipocyte differentiation. These results imply that p38 MAPK and PKC signaling pathways play pivotal roles in the up-regulation of β3-AR mRNA expression during 3T3-L1 preadipocyte differentiation.

### 3.3. Knockdown of β3-AR Vastly Reduces Lipid Accumulation and TG Content during 3T3-L1 Preadipocyte Differentiation

**Figure 3:**
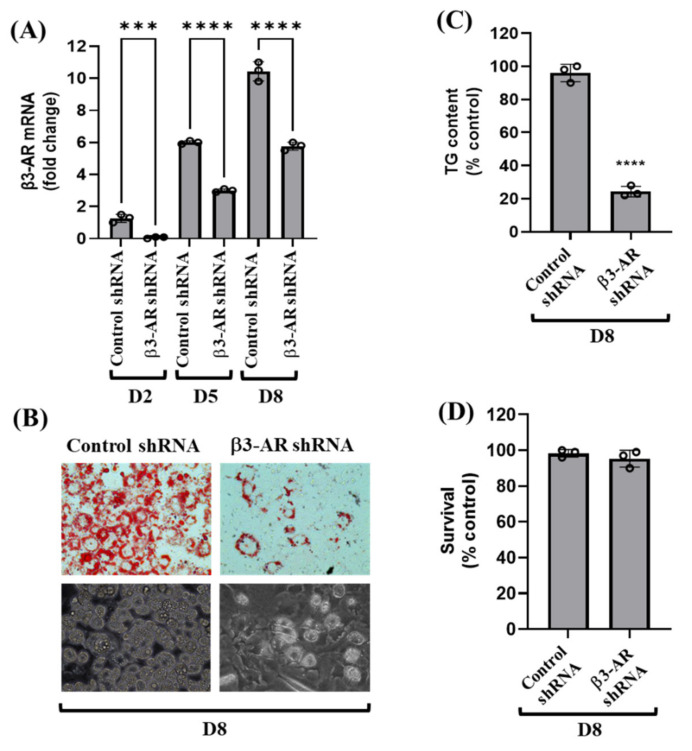
Effects of gene silencing of β3-AR on the expression of β3-AR mRNA, lipid accumulation, TG content, and viability during 3T3-L1 preadipocyte differentiation. (**A**) 3T3-L1 preadipocytes were stably transfected with 100 pM of control or β3-AR shRNA. Control or β3-AR shRNA-transfected cells were differentiated with an induction medium containing MDI, insulin, and FBS, and harvested at day 0 (D0), D2, D5, and D8, respectively. At each time point, total cellular RNA was extracted and analyzed by real-time qPCR with respective primers. Error bars are indicated as mean ± SD. *** *p* < 0.001; **** *p* < 0.0001; calculated by one-way ANOVA with Sidak’s multiple comparison test. (B-D) Control or β3-AR shRNA-transfected cells were differentiated with an induction medium containing MDI, insulin, and FBS, and harvested at D8. Lipid droplets (LDs) accumulation in control or β3-AR shRNA-transfected cells on D8 was measured by Oil Red O staining (upper panels) and by phase-contrast image (lower panels) (**B**). Cellular TG content in control or β3-AR shRNA-transfected cells on D8 was analyzed by AdipoRed assay (**C**). In (**C**), data are mean ± SE of three independent experiments, each performed in triplicate. **** *p* < 0.0001 vs. control (D8). (**D**) The number of live cells in control or β3-AR shRNA-transfected cells on D8 was counted by trypan blue dye exclusion. In (**D**), data are mean ± SE of three independent experiments, each performed in triplicate.

Using β3-AR shRNA transfection, we next sought to directly explore the role of β3-AR in 3T3-L1 preadipocyte differentiation. As shown in Figure 3A, compared with control shRNA-transfected 3T3-L1 cells, there were much lower mRNA expression levels of β3-AR in β3-AR shRNA-transfected cells on D2, D5, and D8 of the cell differentiation, pointing out the β3-AR shRNA transfection efficiency. Importantly, as shown in Figure 3B (upper panels), knockdown of β3-AR led to strong inhibition of lipid accumulation in 3T3-L1 preadipocyte differentiation on D8. The β3-AR-mediated lipid-reducing effect on D8 of 3T3-L1 preadipocyte differentiation was also confirmed by light microscopy (Figure 3B, lower panels). Moreover, knockdown of β3-AR caused a strong reduction of TG content in 3T3-L1 preadipocyte differentiation on D8 (Figure 3C). However, as shown in Figure 3D, results of cell count analysis revealed that knockdown of β3-AR had no cytotoxicity on D8 of 3T3-L1 preadipocyte differentiation. These results indicate that β3-AR plays a positive role in lipid accumulation in 3T3-L1 preadipocyte differentiation.

### 3.4. Knockdown of β3-AR Leads to Decreased Expression and Phosphorylation Levels of C/EBP-α, PPAR-γ, and STAT-5 during 3T3-L1 Preadipocyte Differentiation

**Figure 4:**
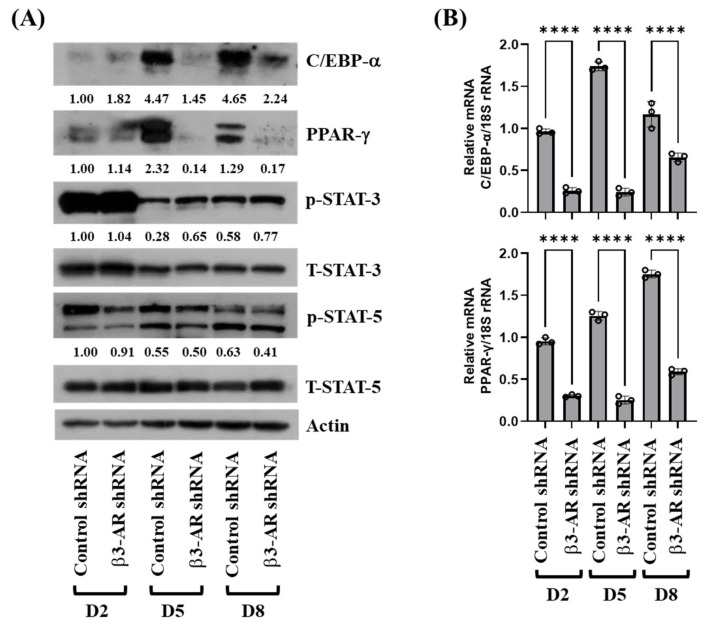
Effects of gene silencing of β3-AR on expression and phosphorylation of C/EBP-α, PPAR-γ, STAT-3, and STAT-5 during 3T3-L1 preadipocyte differentiation. (**A**) 3T3-L1 preadipocytes were stably transfected with 100 pM of control or β3-AR shRNA. Control or β3-AR shRNA-transfected cells were differentiated with an induction medium containing MDI, insulin, and FBS, and harvested at day 0 (D0), D2, D5, and D8, respectively. At each time point, whole-cell lysates were prepared and analyzed by immunoblot analysis with respective antibodies. Relative intensities were measured by ImageJ software (version 1.8.0; National Institutes of Health, Bethesda, MD, USA). (**B**) 3T3-L1 preadipocytes were stably transfected with 100 pM of control or β3-AR shRNA. Control or β3-AR shRNA-transfected cells were differentiated with an induction medium containing MDI, insulin, and FBS, and harvested at day 0 (D0), D2, D5, and D8, respectively. At each time point, total cellular RNA was prepared and analyzed by real-time qPCR with respective primers. Error bars are indicated as mean ± SD. **** *p* < 0.0001; calculated by one-way ANOVA with Sidak’s multiple comparison test.

To elucidate the molecular mechanisms underlying the β3-AR-mediated lipid-reducing effect, we next examined the effects of β3-AR knockdown on expression and/or activity (phosphorylation) of adipogenic transcription factors (C/EBPs, PPARs, and STATs) during the differentiation of 3T3-L1 preadipocytes into adipocytes. As shown in Figure 4A,B, knockdown of β3-AR resulted in strong down-regulation of C/EBP-α and PPAR-γ expressions at the protein and mRNA levels on D5 and D8 of 3T3-L1 preadipocyte differentiation. Knockdown of β3-AR also led to a slight decrease in phosphorylation levels of STAT-5 on D2. However, knockdown of β3-AR had no effects on phosphorylation levels of STAT-3 on D2, D5, and D8 of 3T3-L1 preadipocyte differentiation. Knockdown of β3-AR did not affect total protein levels of STAT-3, STAT-5, and control actin under these experimental conditions. These results suggest that the knockdown of β3-AR regulates the adipogenesis process by downregulation of C/EBP-α and PPAR-γ in adipocyte cells.

### 3.5. Knockdown of β3-AR Results in Down-Regulation of FASN, Perilipin A, and Leptin Expressions during 3T3-L1 Preadipocyte Differentiation

**Figure 5:**
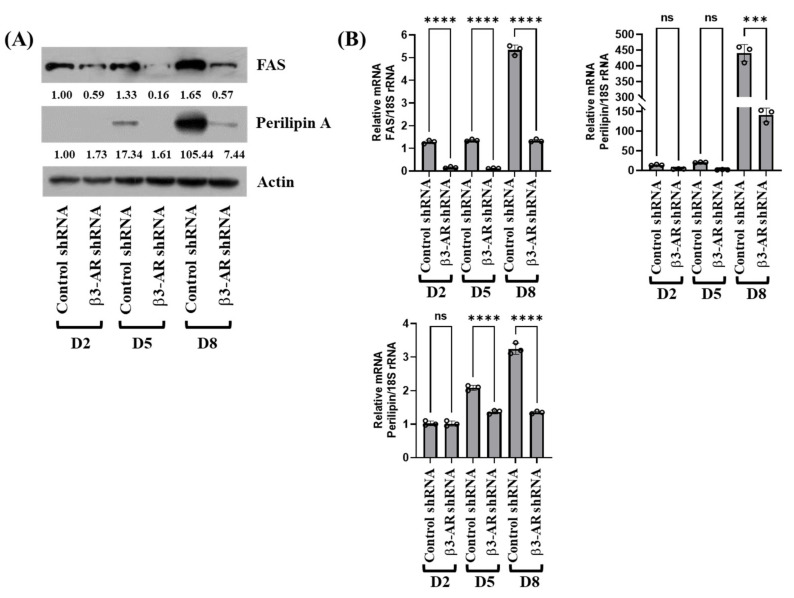
Effects of gene silencing of β3-AR on the expression of FASN, perilipin A, and leptin at the protein and mRNA levels during 3T3-L1 preadipocyte differentiation. (**A**) 3T3-L1 preadipocytes were stably transfected with 100 pM of control or β3-AR shRNA. Control or β3-AR shRNA-transfected cells were differentiated with an induction medium containing MDI, insulin, and FBS, and harvested at day 0 (D0), D2, D5, and D8, respectively. At each time point, whole-cell lysates were prepared and analyzed by immunoblot analysis with respective antibodies. Relative intensities were measured by ImageJ software (version 1.8.0; National Institutes of Health, Bethesda, MD, USA). (**B**) 3T3-L1 preadipocytes were stably transfected with 100 pM of control or β3-AR shRNA. Control or β3-AR shRNA-transfected cells were differentiated with an induction medium containing MDI, insulin, and FBS, and harvested at day 0 (D0), D2, D5, and D8, respectively. At each time point, total cellular RNA was prepared and analyzed by real-time qPCR with respective primers. Error bars are indicated as mean ± SD. *** *p* < 0.001; **** *p* < 0.0001; ns, not significant, calculated by one-way ANOVA with Sidak’s multiple comparison test.

Next, we determined whether β3-AR knockdown alters expression levels of FASN, an enzyme responsible for fatty acid synthesis [24], perilipin A, a lipid droplet-binding and stabilizing protein, and leptin, adipokines, such as leptin, that involve in the pathogenesis of obesity and type 2 diabetes [25], during 3T3-L1 preadipocyte differentiation. As shown in Figure 5A, β3-AR knockdown largely reduced protein expression levels of FASN and perilipin A on D5 and D8 of 3T3-L1 preadipocyte differentiation. Knockdown of β3-AR, however, did not influence expression levels of control actin protein under these experimental conditions. Results of RT-PCR and qPCR, as shown in Figure 5B, further confirmed that β3-AR knockdown greatly suppressed mRNA expression levels of FASN and perilipin A on D5 and D8 of 3T3-L1 preadipocyte differentiation. In addition, there were much fewer mRNA expression levels of leptin in β3-AR shRNA-transfected 3T3-L1 cells on D5 and D8 of differentiation than those in control shRNA-transfected cells. Altogether, these results indicate that β3-AR knockdown reduces both mRNA and protein expression of fatty acid synthesis during adipogenesis.

## 4. Discussion

β3-AR is regarded as a potential anti-obesity target, given that it is expressed predominantly in white and brown adipocytes cells and its selective agonists have lipid-lowering effects by inducing lipolysis and thermogenesis. However, the expression regulation and adipogenic role of β3-AR in white adipocytes remain unclear. Here, we reported that β3-AR expression is highly elevated in differentiating 3T3-L1 preadipocytes in p38 MAPK and PKC-dependent manners. The knockdown of β3-AR resulted in a significant reduction of lipid accumulation as well as a reduction in the TG content in differentiating 3T3-L1 preadipocytes, mediated through modulation of the expression and phosphorylation levels of C/EBP-α, PPAR-γ, STAT-5, FASN, and perilipin A.

As of now, how β3-AR expression is regulated during white preadipocyte differentiation remains unclear. Previous studies have shown the expression of β3-AR in white adipocyte tissue (WAT), but the signals were higher in infant brown adipocyte tissue [26]. As both RT-PCR and RNA-Seq confirm the very low expression of the β3-AR in human WAT depots [27], there might be minor subpopulations of these cells in human adipocytes. The majority of these studies, however, were conducted using β3-AR agonists such as CL316243, which have little effect on the human β3-adrenoceptor. Through initial experiments, we demonstrated that β3-AR expression at mRNA levels is highly induced in a time-dependent fashion compared to protein expression during the differentiation of 3T3-L1 preadipocyte into adipocytes. Of note, data of pharmacological inhibition studies showed that the mRNA expression of β3-AR during 3T3-L1 preadipocyte differentiation is greatly repressed in the presence of p38 MAPK or PKC inhibitor. These results advocate that the activities of p38 MAPK and PKC are crucial for the induction of β3-AR mRNA expression during 3T3-L1 preadipocyte differentiation.

It has been well documented that the constitutive activation of β3-AR induces the appearance of brown/beige adipocytes in different adipose tissue depots via adipogenesis [28]. However, the function of β3-AR in 3T3-L1 preadipocyte differentiation remains elusive. Strikingly, in the current study, gene silencing of β3-AR results in less lipid accumulation and TG content during 3T3-L1 preadipocyte differentiation, which illustrates a positive role of β3-AR in lipid accumulation during 3T3-L1 preadipocyte differentiation. A large body of evidence strongly supports the pivotal role of C/EBP-α, PPAR-γ, STAT-3, and STAT-5 in preadipocyte differentiation [15,16,29,30,31]. Up to date, β3-AR regulation of C/EBP-α, PPAR-γ, STAT-3, and STAT-5 during white preadipocyte differentiation is unknown. Of interest, we herein observed that gene silencing of β3-AR leads to the reduced expression of C/EBP-α and PPAR-γ at both protein and mRNA levels in differentiating 3T3-L1 cells, addressing that β3-AR knockdown-mediated C/EBP-α and PPAR-γ down-regulation in differentiating 3T3-L1 cells is due to their transcriptional repression. Additionally, β3-AR knockdown also leads to partial inhibition of STAT-5 phosphorylation without altering total STAT-5 protein levels at the early stage of 3T3-L1 preadipocyte differentiation (D2), suggesting that β3-AR knockdown partially inhibits phosphorylation of pre-existed STAT-5 without de novo protein synthesis. Taken together, these results suggest that β3-AR’s lipid-accumulative effect on differentiating 3T3-L1 cells is closely linked to increased expression and phosphorylation levels of C/EBP-α, PPAR-γ, and STAT-5.

Adipocyte differentiation also involves lipogenesis and LDs stabilization [29,30]. FASN is a lipogenic enzyme responsible for the synthesis of fatty acids [11,24]. Perilipin A, a protein that binds to and stabilizes the newly synthesized LDs, is necessary for lipid accumulation and storage [25,31]. It has not been studied yet how β3-AR regulates FASN and perilipin A during white preadipocyte differentiation. In the present study, gene silencing of β3-AR further leads to strong down-regulation of FASN and perilipin A at both protein and mRNA levels at the middle (D5) and late (D8) stage of 3T3-L1 preadipocyte differentiation. It is thus likely that β3-AR’s lipid-accumulative effects on differentiating 3T3-L1 cells are also attributable to the increased expression of FASN and perilipin A. Accordingly, adipocytes express and secrete an array of adipokines, such as leptin, that involve in the pathogenesis of obesity and type 2 diabetes [32]. We demonstrated that the mRNA of leptin is highly up-regulated on D5 and D8 of 3T3-L1 preadipocyte differentiation; however, knockdown of β3-AR results in strong repression of leptin mRNA expression. These results indicate that β3-AR expression is crucial for leptin expression during 3T3-L1 preadipocyte differentiation. Given that the expression of FASN, perilipin A, and leptin is largely influenced by PPAR-γ in preadipocyte differentiation, it is speculative that β3-AR knockdown-mediated inhibition of FASN, perilipin A, and leptin expression in differentiating 3T3-L1 cells is due to PPAR-γ down-regulation.

## 5. Conclusions

We demonstrate that β3-AR expression is highly up-regulated in p38 MAPK and PKC-dependent manners. The up-regulated β3-AR plays a crucial role in lipid accumulation or storage in differentiating 3T3-L1 cells, mediated through modulation of expression and phosphorylation levels of C/EBP-α, PPAR-γ, STAT-5, FASN, and perilipin A. The present findings suggest that inhibitors of β3-AR expression or β3-AR antagonist(s) may be used as potential preventive and therapeutics against obesity.

## Data Availability

The data presented in this study are available in this article.
